# Successful long-term survival following lipiodol chemoembolization for hepatocellular carcinoma in Alagille syndrome: A case report

**DOI:** 10.1016/j.radcr.2025.04.125

**Published:** 2025-05-20

**Authors:** Imad Akasbi, Ismail Chaouche, Nizar El Bouardi, Amal Akammar, Hajar O Chahdi, Ismail Zerrari, Maria Lahlali, Hakima Abid, Badr Alami, Nada Lahmidani, Meryem Boubbou, Ibrahimi Sidi adil, Mustapha Maaroufi, MY Youssef Alaoui Lamrani

**Affiliations:** a Adult Radiology Department*,* CHU Hassan II*,* Sidi Mohammed Ben Abdellah University*,* Fez*,* Morocco; b Radiology Department*,* Mother and Child Hospital, CHU Hassan II*,* Sidi Mohammed Ben Abdellah University*,* Fez*,* Morocco; c Gastroenterology Department*,* CHU Hassan II*,* Sidi Mohammed Ben Abdellah University*,* Fez*,* Morocco; d Digestive Research Laboratory*,* CHU Hassan II*,* Sidi Mohammed Ben Abdellah University*,* Fez*,* Morocco; e Department of Radiology and Clinical Imaging, The Clinical Neurosciences Laboratory, Faculty of medicine and Pharmacy, University of Fez*,* Fez*,* Morocco; f Department of Biophysics and Clinical MRI Methods, Faculty of Medicine and Pharmacy, University of Fez, Fez, Morocco

**Keywords:** HCC, Lipiodol chemoembolization, Alagille syndrome

## Abstract

Alagille syndrome is a rare genetic disorder causing multisystem complications, including biliary abnormalities, chronic liver disease, and rarely, hepatocellular carcinoma (HCC), which is difficult to manage due to liver dysfunction and vascular abnormalities. We report a 29-year-old female, diagnosed with Alagille syndrome in infancy, who developed infiltrative HCC at age 20 and underwent lipiodol-based transarterial chemoembolization (TACE) in 2015, with a second session in 2016 for recurrence. Remarkably, she has achieved an exceptional 9-year survival post-treatment with stable imaging and no recurrence, a highly uncommon outcome for HCC in Alagille syndrome, highlighting TACE’s potential as an effective palliative treatment for HCC in Alagille syndrome, particularly for patient's ineligible for liver transplantation, and underscoring the need for further studies on locoregional therapies in this rare population.

## Introduction

Alagille syndrome, a rare autosomal dominant genetic disorder, is marked by multisystem involvement, notably chronic liver disease due to bile duct paucity [[1], [2], [3]]. This case was selected due to the rarity of HCC in Alagille syndrome and the exceptional long-term survival observed following chemoembolization. While liver transplantation remains the definitive treatment, many patients are ineligible due to limited donor availability and surgical risks [[3], [4], [5]]. This case provides valuable insight into the potential of transarterial chemoembolization as a long-term disease control strategy in such patients [4,5]. The patient’s favorable outcome underscores the importance of a tailored, multidisciplinary approach, with regular monitoring and coordinated care among hepatologists, oncologists, and radiologists to optimize outcomes and enhance quality of life in complex cases [[2], [3], [4], [5]].

## Case description

We report the case of a 29-year-old woman with Alagille syndrome, diagnosed at 40 days of age based on neonatal cholestatic jaundice, characteristic facial phenotype, posterior embryotoxon, and growth retardation with no family history. Since infancy, she has been regularly monitored for chronic cholestasis, which progressed to infiltrative hepatocellular carcinoma (HCC) diagnosed in 2015 at age 20, on a cirrhotic liver secondary to her genetic condition, her initial alpha-fetoprotein (AFP) was elevated at 282 ng/mL.

**Fig. 1:**
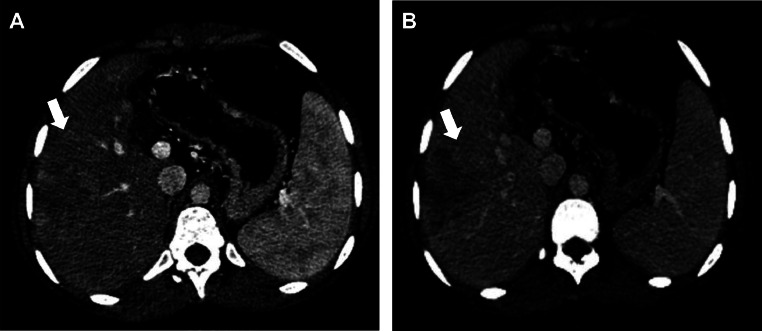
Axial CT scan showing a multinodular infiltrative HCC in segments VI and VII; hypervascular lesions on arterial phase (A) with washout in portal phase (B) (white arrow).

**Fig. 2:**
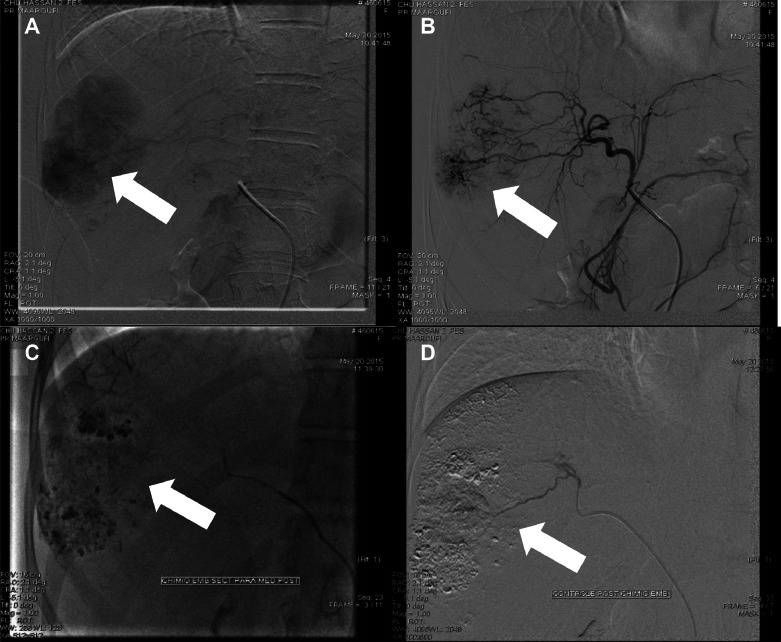
First TACE (2015): Lipiodol chemoembolization of hepatic segments VI and VII, (A, B, C, D).

**Fig. 3:**
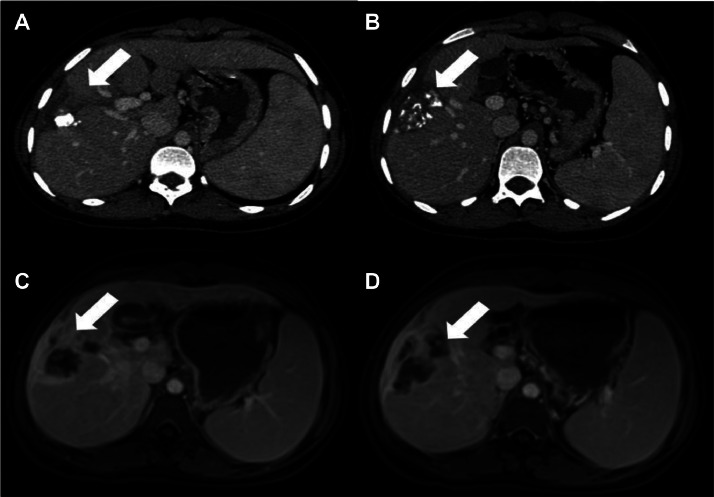
Post first TACE, axial CT scan (A, B) axial injected T1 (C, D) showing a dense and heterogeneous Lipiodol retention in treated lesions.

**Fig. 4:**
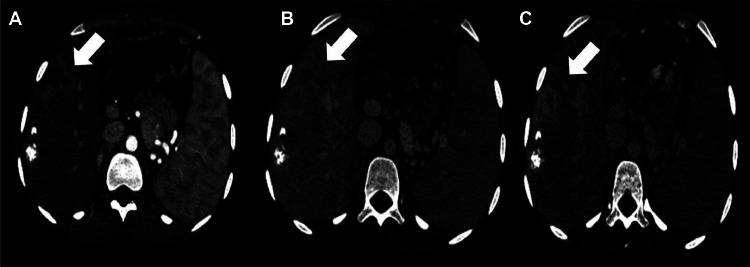
Axial CT scan showing a residual hypervascular tumor in segments VI–VII (A); reappearance of enhancement suggestive of viable tumor tissue.

**Fig. 5:**
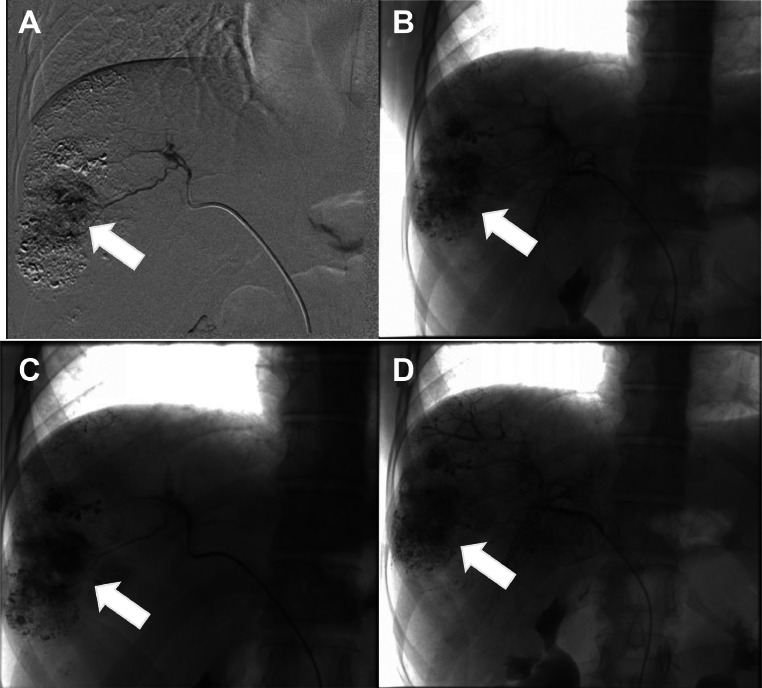
Second TACE (2016): Lipiodol chemoembolization of hepatic segments VI and VII (A, B, C, D).

**Fig. 6:**
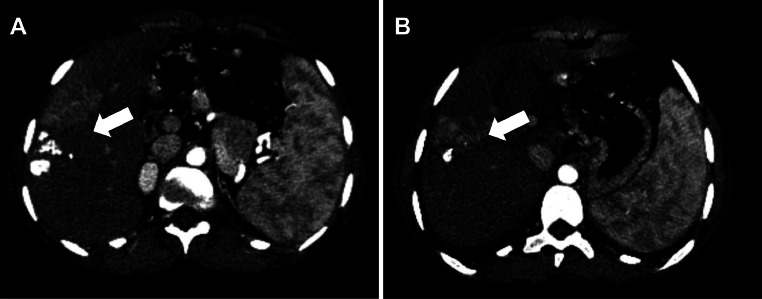
Axial CT scan showing a complete Lipiodol uptake in embolized areas; absence of new enhancing lesions; stasis achieved in targeted artery (white arrow).

A contrast-enhanced CT confirmed liver lesions in segments VI and VII, suggestive of HCC (Fig. 1). After multidisciplinary tumor board discussion, the patient underwent transarterial chemoembolization (TACE) in May 2015 (Fig. 2), followed by a second session in 2016 due to recurrence (Fig. 5) Fig. 3, Fig. 4, Fig. 5, Fig. 6.

Since then, the patient has been closely followed with clinical assessments, liver function tests, tumor marker monitoring, and periodic imaging. Her biological profile has remained stable, with key findings including:

■ Hemoglobin levels of 12.9-14.2 g/dL.
■ Mild thrombocytopenia (platelet counts: 126,000-144,000/mm³).
■ Prothrombin activity of 71%-86%.
■ Mild to moderate transaminase elevation.
■ Serum albumin levels of 42-45 g/L.
■ Persistent elevation of total bilirubin (20-37 mg/L) and direct bilirubin (up to 30 mg/L).
■ AFP levels consistently low (13-15 ng/mL), with no concerning trends.

**Fig. 7:**
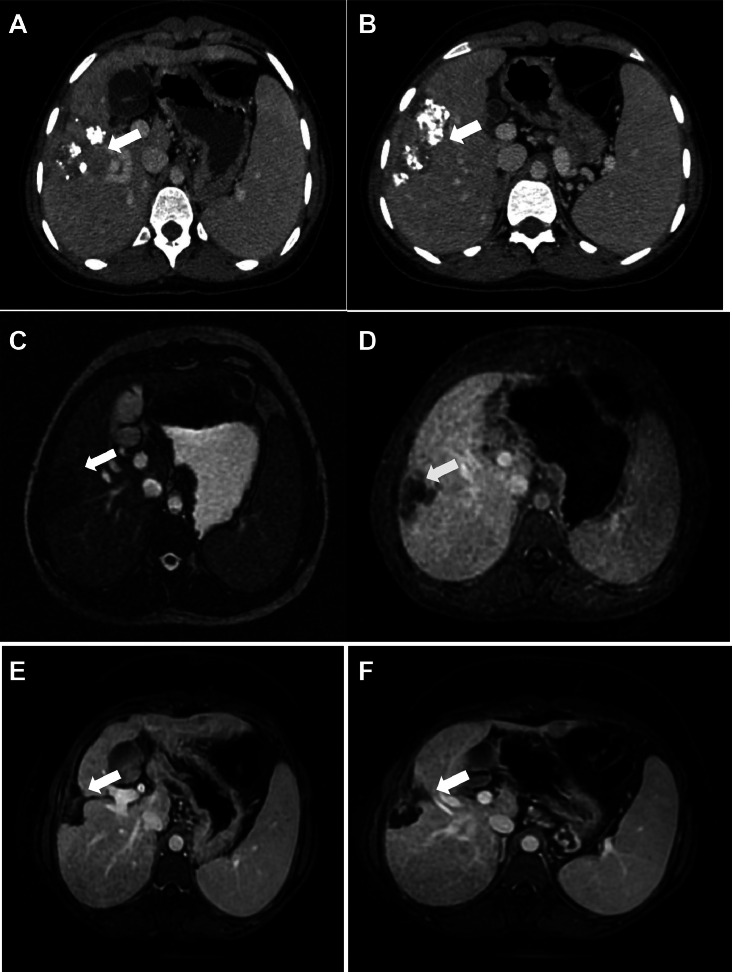
Long-term follow-up (2017-2024): Axial CT scans (A, B), MRI sequences; 2D FIESTA (C) and T1 GADO (D, E, F) showing a persistent lipiodol deposition in treated areas; no evidence of viable tumor; signs of capsular retraction; no recurrence after second embolization (white arrow).

**Fig. 8:**
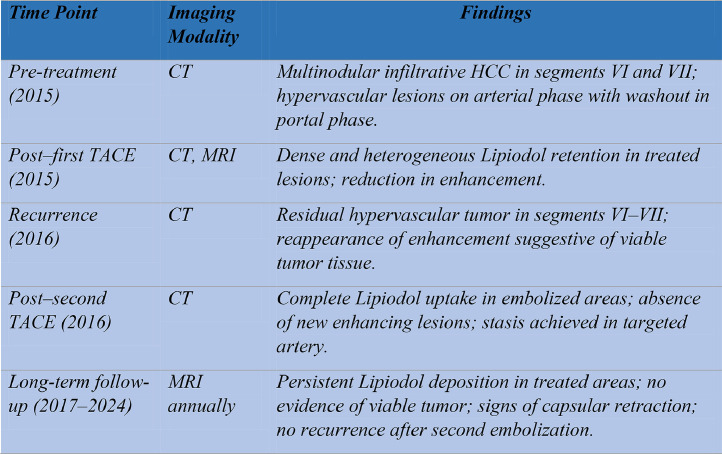
Longitudinal summary of imaging findings and treatment response over 9 years.

Follow-up hepatic MRIs and angio-CTs showed no contrast enhancement in the embolized HCC lesions and with no recurrence or significant progression demonstrating a good therapeutic response (Fig. 7), over 9 years. Fig. 8.

In 2021, upper gastrointestinal endoscopy revealed esophageal varices, indicating portal hypertension, and a histologically confirmed Barrett’s esophagus segment without dysplasia. Beta-blocker therapy was considered but deferred pending follow-up endoscopy, which the patient occasionally declined.

Clinically, the patient has remained in good condition, with no evidence of jaundice, ascites, or hepatic encephalopathy. Although adherence to medical appointments has been inconsistent, her liver function has been preserved, with a Child-Pugh score of A5–A6 when complete data were available. Biochemical and clinical findings consistently indicate a favorable response to TACE.

This case underscores the long-term management of HCC in Alagille syndrome, effectively controlled through TACE and multidisciplinary surveillance.

## TACE procedure details

In our setting, doxorubicin is the primary agent for HCC embolization. An emulsion of 1 mL Ultravist® per 20 mg doxorubicin is prepared, with 50-75 mg/m² of doxorubicin (Adriblastine®) diluted in 20 ml of 5% glucose serum. The mixture is created using a pumping technique with 2 syringes connected by a 2-way valve, then combined with 10 ml of iodized oil (Lipiodol®). The emulsion is injected through a catheter into the tumor-feeding vessels.

During the procedure, catheterization of the celiac trunk revealed an arterial vascular blush in segments VI and VII, linked to branches of the right hepatic artery arising from a quadrifurcation. Selective angiography confirmed tumor vascularization via the right hepatic artery, which was hyperselectively catheterized using a 2.3 Fr microcatheter. A mixture of 50% Lipiodol and 50% doxorubicin was injected until complete stasis was achieved. Postprocedural imaging confirmed dense Lipiodol accumulation within the tumor, suggesting successful embolization.

## Discussion

The development of HCC in Alagille syndrome is caused by fibrotic changes, genetic instability in hepatocytes and oncogenic mutations [[1], [2], [3]]. Furthermore, in the context of genetic predisposition, mutations in the JAG1 or NOTCH2 genes can contribute directly to carcinogenesis by perturbing cell signaling pathways [[3], [4], [5], [6]]. Nonspecific markers such as bilirubin and the presence of xanthomas have been identified as potential predictors of severe liver damage with an increased risk of malignancy [1,2,4,5].

The management of Alagille syndrome and its complications requires a multidisciplinary approach. Regular monitoring of liver function and imaging is essential for early detection of malignancies. Liver transplantation remains the definitive treatment for end-stage liver disease and HCC in this syndrome, offering better survival outcomes. However, challenges such as organ availability and per-operative risks need to be surmounted [[3], [4], [5]].

HCC in Alagille syndrome remains poorly studied, with only a handful of reported cases. In a retrospective study by Pham et al., 1 patient with Alagille syndrome underwent chemoembolization with good response, though liver transplantation was ultimately required. Geramizadeh et al. described another case of pediatric HCC in Alagille syndrome, where chemoembolization was performed twice, achieving a durable clinical and radiological response [[7], [8], [9]] Our patient's case stands out due to the exceptional long-term survival of 9 years without liver transplantation, highlighting the potential role of repeated chemoembolization as a bridging or palliative strategy when transplant is not feasible.

Emerging therapies for Alagille syndrome target the underlying genetic and molecular mechanisms, especially the Notch signaling pathway (Kohut et al. [3]). Gene-based treatments and small molecules are being developed to address JAG1 or NOTCH2 mutations. Novel agents also aim to improve bile acid metabolism and reduce fibrosis (Ayoub et al. [5]). These approaches may help manage cholestasis and prevent liver damage. Advances in genotype-phenotype understanding support more personalized care. Ongoing trials are essential to confirm long-term safety and efficacy [[3], [4], [5], [6]].

A neoadjuvant therapy combining immunotherapy, anti-angiogenic therapy acting in synergy with radiotherapy (to stimulate an immune response) has been described by Li et al. [10] as an innovative and radical combined therapeutic approach to treat particularly highly infiltrative and massive HCC. Indeed, the authors report that this combination enabled a complete response in an HCC patient with an overall survival of 36 months, which may outperform other conventional treatment approaches [10].

## Conclusion

HCC is an aggressive complication of Alagille syndrome, and treatment options remain limited. This case highlights the feasibility of long-term disease control with lipiodol chemoembolization, even in patients who are ineligible for liver transplantation. While chemoembolization is not curative, it can serve as a valuable palliative or bridging therapy in carefully selected patients. Future studies should focus on evaluating the safety and efficacy of locoregional therapies in this rare patient population to optimize outcomes and expand treatment options.

This case highlights the potential of lipiodol chemoembolization as an effective palliative treatment for HCC in Alagille syndrome, particularly in patients who are ineligible for liver transplantation. Long-term survival, as observed in this case, underscores the need for further studies on locoregional therapies in this rare population.

We emphasize the need for multidisciplinary collaboration between hepatologists, oncologists and interventional radiologists to optimize outcomes. Future studies and research should focus on larger populations and move towards comparative studies to accurately evaluate the safety and efficacy of chemoembolization and other locoregional therapies in HCC associated with Alagille syndrome. It will be essential and crucial to develop personalized therapeutic strategies to meet the challenges presented by this multisystemic pathology and improve patients' prognosis and quality of life.

In our case, chemoembolization was the best therapeutic approach, with stability over a period of 09 years, combined with radiological and biological monitoring, improving the patient's quality of life.

## Patient consent

Written informed consent was obtained from the patient for the publication of this case report and any accompanying images. The patient has been assured that all personal information will remain confidential and that identifying details will not be disclosed.

The patient was also informed that this publication is for scientific purposes and has the right to withdraw consent at any time without any impact on their medical care.
